# Hemodynamic collapse due to the stenosis of four pulmonary veins in a patient with mediastinal tumor

**DOI:** 10.1002/ccr3.924

**Published:** 2017-04-05

**Authors:** Kentaro Yoshida, Fumie Fujisawa, Hiroshi Kojima, Noriyuki Takeyasu

**Affiliations:** ^1^ Department of CardiologyFaculty of MedicineUniversity of TsukubaTsukubaJapan; ^2^ Department of CardiologyIbaraki Prefectural Central HospitalKasamaJapan; ^3^ Department of Medical OncologyIbaraki Prefectural Central HospitalKasamaJapan

**Keywords:** Cardiogenic shock, lymphoma, mediastinal tumor, pulmonary vein stenosis

## Abstract

Cardiogenic shock can occur due to compression of the four pulmonary veins and the left atrium by a mediastinal tumor. Steroid infusion can be a temporary alternative therapy before obtaining a definite diagnosis and performing an intervention with stents to dilate the pulmonary veins.

An 81‐year‐old man who had undergone left upper lobectomy due to squamous cell carcinoma 7 years ago was referred to our emergency department because of severe dyspnea. Physical examination showed signs of cardiogenic shock and a chest X‐ray revealed lung congestion. An echocardiographic evaluation revealed decreased left atrial cavity size due to a compressive tumor, and ECG‐gated cardiac computed tomography (CT) was subsequently performed that revealed a large tumor (40 mm minor axis diameter) in the posterior mediastinum and compression of the left atrium and pulmonary veins (PVs) (Fig. 1A). The left upper PV was a blind cap after the lobectomy, and occlusion of the other three PVs was impending. Pericarditis associated with the metastasis or invasion of tumor cells was also suggested. We scheduled an emergent intervention to dilate the PVs with self‐expandable stents. However, 1 day after an infusion of dexamethasone 12 mg to improve the patient's symptoms, his vital signs became stable, and the dyspnea promptly disappeared. Of interest, a CT scan repeated 8 days after the beginning of dexamethasone infusion revealed a dramatical decrease in tumor size and resolution of the PV stenosis and pericarditis (Fig. 1B). Because of the very good response to the steroid therapy, lymphatic malignancy was a more likely diagnosis than metastasis of squamous cell carcinoma of the lung. The patient was referred to the oncology department for further management.

**Figure 1:**
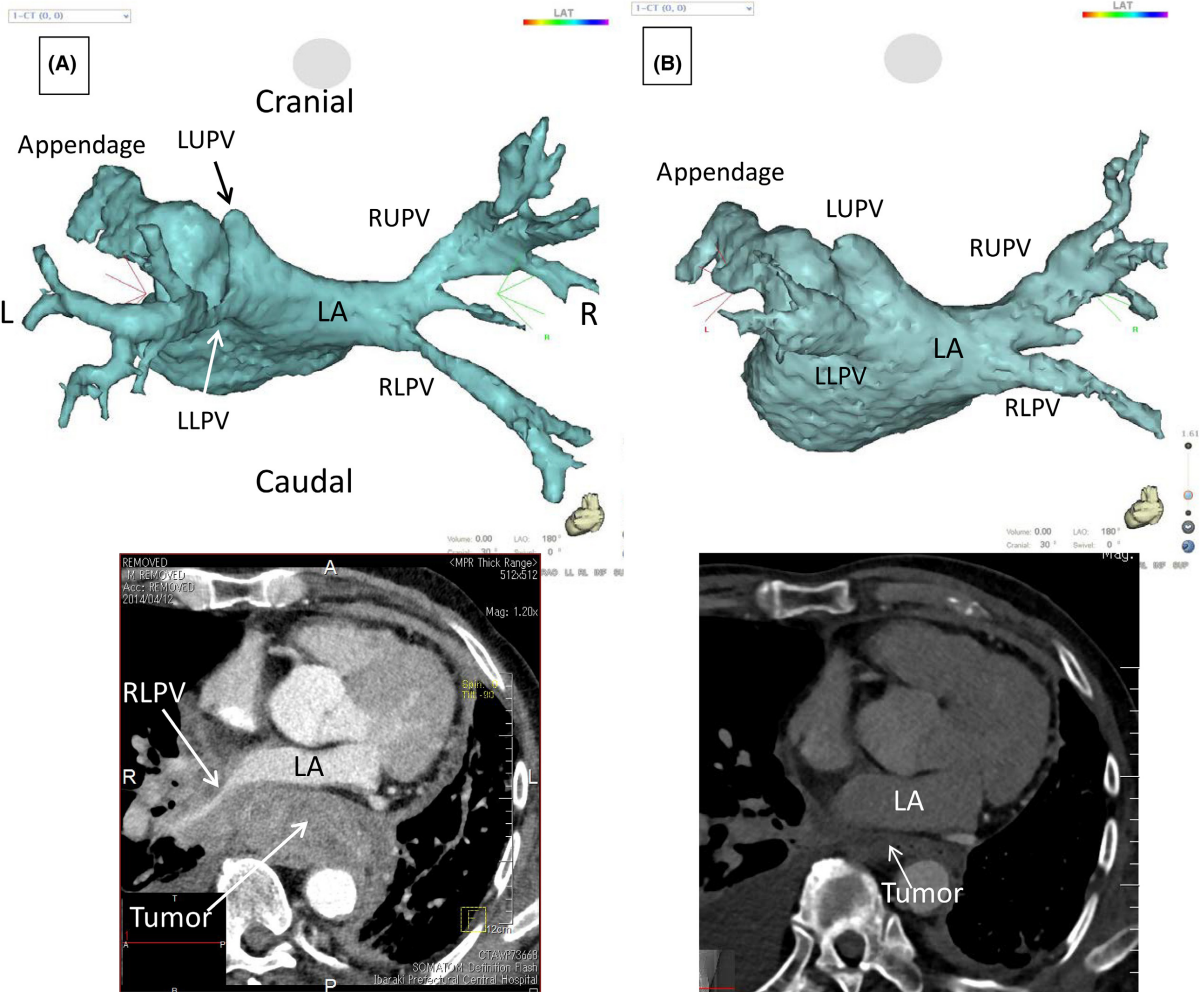
Three‐dimensional reconstruction of the left atrium and pulmonary veins from the ECG‐gated cardiac computed tomography scan. L, left; LA, left atrium; LLPV, left lower pulmonary vein; LUPV, left upper pulmonary vein; R, right; RLPV, right lower pulmonary vein; RUPV, right upper pulmonary vein.

Single PV stenosis due to a compressive or invasive tumor was previously reported in a few case reports 1, 2. It was notable that the present patient suffered from hemodynamic collapse due to the impending occlusion of all three functional PVs and that the tumor size unexpectedly decreased immediately after steroid infusion. From the results of the present case, physicians should consider the possibility that dislodgement or dropout of stents can occur when chemotherapy dramatically decreases tumor volume and the severe stenosis in the PVs is resolved within a short time after stenting.

## Authorship

KY and FF: equally contributed to the conception of the work. KY: wrote the first draft of the manuscript. All authors: revised it critically and approved the final version to be published.

## Conflict of Interest

None declared.
